# Construction and validation of a checklist for the management of totally implanted catheters in children

**DOI:** 10.1590/0034-7167-2023-0447

**Published:** 2024-09-20

**Authors:** Giselle Vieira de Souza, Isabella Pavarine de Souza, Maria Paula Custodio Silva, Silmara Elaine Malaguti Toffano, Divanice Contim

**Affiliations:** I Universidade Federal do Triângulo Mineiro. Uberaba, Minas Gerais, Brazil; II Universidade Federal do Triângulo Mineiro, Rede Brazileira de Serviços Hospitalares, Hospital de Clínicas. Uberaba, Minas Gerais, Brazil

**Keywords:** Nursing Care, Validation Study, Vascular Access Devices, Pediatric Nursing, Checklist, Atención de Enfermería, Estudio de Validación, Dispositivos de Acceso Vascular, Enfermería Pediátrica, Lista de Verificación

## Abstract

**Objectives:**

to construct and validate the content of a checklist for the management of totally implanted catheters in hospitalized children and adolescents.

**Methods:**

methodological research conducted from October 2021 to December 2022 in two stages: development of the instrument with care guidelines and content validation of the checklist. The instrument, containing 23 items presented in Likert format, was evaluated online by specialists in two rounds. The Content Validity Index was applied, considering indices above 0.8 as valid.

**Results:**

the final checklist included four domains and 22 checklist items, validated with a Content Validity Index of 0.98. The overall evaluation of the instrument presented a global score of 9.9.

**Conclusions:**

the validation and application of instruments that standardize procedures, in addition to supporting professionals, promote autonomy and quality of care for children and adolescents using this device.

## RESUMEN

**Objetivos:**

construir y validar el contenido de una lista de verificación para el manejo del catéter totalmente implantado en niños y adolescentes hospitalizados.

**Métodos:**

investigación metodológica realizada en el período de octubre de 2021 a diciembre de 2022, en dos etapas: elaboración del instrumento con directrices para el cuidado y validación de contenido de la lista de verificación. El instrumento, que contiene 23 ítems presentados en formato Likert, fue evaluado en línea por especialistas en dos rondas. Se aplicó el Índice de Validez de Contenido, considerando válidos los índices superiores a 0,8.

**Resultados:**

la lista final contempló cuatro dominios y 22 ítems de verificación, siendo validada con un Índice de Validez de 0,98. La evaluación general del instrumento presentó una calificación global de 9,9.

**Conclusiones:**

la validación y aplicación de instrumentos que estandarizan los procedimientos, además de respaldar al profesional, promueven la autonomía y calidad de la asistencia al niño y al adolescente en uso de este dispositivo.

## INTRODUCTION

According to the World Health Organization (WHO), approximately 400,000 children and adolescents are diagnosed with childhood cancer each year. This is a heterogeneous age group with rare diseases that emerge between birth and 19 years of age^(1)^. Childhood cancers originate in embryonic tissues that undergo mutations resulting from the uncontrolled division of abnormal cells. The most common types are leukemia, associated with the lymphatic system; tumors such as neuroblastoma, occurring in multiple locations but especially in the adrenal glands and related to the central nervous system; lymphoma, linked to immune cells; kidney tumors; and bone tumors^(2)^.

In Brazil, in 2023, approximately 7,930 new cases of cancer were registered among children and adolescents, with an estimated risk distribution of 140.50 cases per million for males and 128.87 per million for females. These data are significant, given that about 33% of the Brazilian population falls within this age group^(3)^. Neoplasms are the leading cause of death in children from one year old to the end of adolescence^(3)^.

Advances in the treatment of childhood cancer over the past 50 years have significantly impacted survival rates, with an increase of over 85% in global survival rates^(4-5)^. The development of technologies related to childhood cancer treatment has incorporated the totally implanted central venous catheter (CVC-TI) into healthcare, providing better quality of life for patients. This device provides venous access for the frequent administration of blood components, blood derivatives, parenteral nutrition, antibiotics, and chemotherapeutic agents in patients requiring long-term intravenous therapies (IV)^(6)^.

The insertion of the CVC-TI, compared to other devices, decreases the risk of thrombosis, reduces infection rates, provides greater comfort to the patient, enables outpatient treatment, preserves the peripheral venous system, and maintains the patient’s activity routine, thereby reducing stress and suffering from numerous unsuccessful punctures^(6)^.

The nursing team, especially nurses, are primarily responsible for the manipulation and maintenance of the CVC-TI. According to the regulations of the Federal Nursing Council (COFEN), which stipulate the exclusive role of the nurse, it is the nurse’s responsibility to perform the CVC-TI puncture, administer chemotherapeutic agents, and take care of this device. Therefore, nurses must have knowledge and develop competencies in catheter management, acting safely and providing essential practices during the treatment of patients requiring this device^(7-8)^.

Proper management of the CVC-TI helps reduce costs, prevents occlusions, and lowers the risk of infections associated with healthcare. Thus, it is essential to develop instruments to enhance safety and improve the quality of nursing care provided to patients using this device^(8)^.

Checklists are simple and cost-effective tools that can be applied across various areas of healthcare. They contribute to care management, improve the quality of care provided, and increase patient safety by enabling the delivery of safe and reliable care^(9-10)^. As a structured work tool that encompasses a set of complex items or activities, checklists are used to ensure that the necessary actions and interventions for a given task have been completed^(11)^. Studies indicate that this tool enhances the quality of care and reduces adverse events related to patient safety^(9-11)^.

The importance of developing a checklist for the management of totally implanted catheters in hospitalized children and adolescents becomes evident, as it ensures the provision of quality care. Therefore, it is essential to address the following question: What care procedures for managing a totally implanted central venous catheter in hospitalized children and adolescents should be included in a checklist?

## OBJECTIVES

To construct and validate the content of a checklist for the management of totally implanted catheters in hospitalized children and adolescents.

## METHODS

### Ethical Aspects

To comply with the requirements of Resolution 466/2012 of the National Health Council, which regulates the norms for research involving human subjects, all participants signed the Free and Informed Consent Form. The study was approved by the Research Ethics Committee of the *Hospital de Clínicas da Universidade Federal do Triângulo Mineiro* (HC-UFTM), and the approval statement is attached to this submission.

### Type and Period of Study

This methodological study was conducted from March to November 2022, focusing on the development and validation of a checklist for the management of CVC-TI in hospitalized children and adolescents. The study was divided into two phases: the creation and validation of the instrument, which was analyzed by specialists in pediatric oncology nursing^(12)^. Following the creation of the instrument, the validation process was carried out through an online panel of experts using a combined strategy of content validation and consensus building^(13)^.

### Population and Sample

For the content validation phase, pediatric nursing specialists were selected through a search in the Lattes Curriculum database, using the professional activity filter. Sixty-five nurses who met Fehring’s criteria^(14)^ were selected.

A 15-day deadline was established for the return of the evaluation instruments. At this point, individuals who reported reasons preventing their participation in the study were excluded. Fourteen participants returned the completed instrument in the first round of evaluation. In the second round, the suggested adjustments were made, and the instrument was emailed to the fourteen specialists from the first round, resulting in seven evaluations being returned.

### Methodological Procedures

The instrument was constructed by the researchers based on the Standard Operating Procedure (SOP) for the Totally Implanted Catheter Puncture of HC-UFTM^(15)^, the “Measures for the Prevention of Healthcare-Associated Infections” handbook by the National Health Surveillance Agency (ANVISA)^(16)^, and a study on catheter heparinization and salinization^(17)^.

The first version of the checklist was structured around four domains: Pre-procedure, Procedure - puncture, Post-procedure, and Maintenance. The checklist, named “Checklist for the Management of Totally Implanted Catheters in Hospitalized Children and Adolescents”, was created in HyperText Markup Language (HTML) using Google Forms^®^.

Specialists received an invitation letter via email, explaining the objectives and procedures of the study and requesting their participation. After this phase, potential participants received, through the Google Forms platform, a Free and Informed Consent Form. Upon signing it, they received a link to access a sociodemographic characterization form, including gender, age, length of professional experience, academic background, time working in pediatric oncology nursing, and the checklist to be validated.

To evaluate each listed item, a five-point Likert scale was used, where: (five) equals strongly agree, (four) equals agree, (three) equals neither agree nor disagree, (two) equals disagree, and (one) equals strongly disagree. An optional space for descriptive responses was also included. For the overall analysis of the instrument, the following criteria were considered: usefulness/relevance, consistency, clarity, objectivity, simplicity, feasibility, update, vocabulary, accuracy, instructional sequence of topics; and finally, the global score^(13)^.

### Study Analysis and Statistics

The data obtained were imported from Google Forms^®^ into a Microsoft Excel^®^ database and analyzed based on the Content Validity Index (CVI), which measures the proportion or percentage of experts who agree with specific aspects of the instrument and its items, allowing for an individualized analysis of each item and the instrument as a whole. The CVI considered valid for this study was above 0.8^(18)^. The adjustments and suggestions from the first round were included and submitted in the second round of evaluation. The item-level CVI (I-CVI) was calculated by summing the agreement of items rated as four or five. Items that received a score of one or two were revised or eliminated.

## RESULTS

Of the total specialists who agreed to participate in the study, 14 were part of the sample for the first round and seven for the second round of validation. All were female nurses, with an average age of 40 years, ranging from 28 to 64 years. Among them, 14% had a master’s degree and 86% had a doctorate. Additionally, 64% were faculty members in the field of pediatric nursing, and 26% worked in federal hospitals, with an average experience of six years in pediatric oncology or maternal-infant nursing. In total, 78% had completed a catheter management course and 85% performed CVC-TI punctures.

The first version sent out had 23 items, subdivided into four domains: Pre-procedure, Procedure - puncture, Post-procedure, and Maintenance. All items were evaluated using the Likert scale. Suggestions were considered, and necessary alterations were made.

For all items, agreement was within the established level (CVI > 0.8). The suggestions were considered, changes were made, and the instrument was sent for a new round. Among the suggestions, two questions (6 and 7) were combined, resulting in a final instrument with 22 items. The second round was used to make adjustments to the validated instrument, and its CVI is described in Table 1.

**Table 1:** Second round of content validation of the care checklist for the management of totally implanted catheters in hospitalized children and adolescents by the seven specialists, considering the Content Validity Index, Uberaba, Minas Gerais, Brazil, 2023

Management of Totally Implanted Catheter in Hospitalized Children	CVI
Pre-procedure	
1. Verify patient identification. Check name and ID number.	1.00
2. Explain the procedure to the patient and caregiver.	1.00
3. Sanitize hands with soap and water or 70% alcohol gel using aseptic technique.	1.00
4. Gather materials and place them on a mobile utility table near the patient’s bed.	1.00
5. Sanitize hands with 2% chlorhexidine scrub or an alcohol-based preparation recommended by the institution.	0.97
6. Don personal protective equipment (cap, surgical mask for both professional and patient, disposable gown, and sterile gloves).	0.91
Procedure - Puncture	
7. Perform antisepsis of the reservoir with 0.5% to 2% alcoholic chlorhexidine, using sterile gauze and forceps, in a Z-technique, for 30 seconds. (Repeat 3 times)	0.89
8. Wait until the alcohol solution evaporates from the skin, or dry with sterile gauze.	0.89
9. Place a fenestrated drape or sterile compress at the puncture site.	1.00
10. Stabilize the catheter reservoir between the thumb and index finger of the non-dominant hand.	1.00
11. Puncture the central region of the reservoir, gently inserting the Huber needle at a 90° angle until it touches the bottom.	1.00
12. Aspirate using a 10 ml syringe to withdraw about 2 ml of the solution from the reservoir or until blood reflux is observed.	1.00
13. Test the catheter’s patency. Infuse 10 ml of 0.9% saline solution using a turbulence technique. Clamp the extension after confirming patency.	1.00
Post-procedure	
14. Apply an occlusive dressing with sterile polyurethane transparent film or sterile gauze and adhesive tape.	1.00
15. Change the dressing according to the type of covering used. For sterilized transparent polyurethane film, change every 7 days. For dressing with sterile gauze and adhesive tape, change within 48 hours. Change immediately if the dressing is loose, wet, or soiled.	1.00
16. Label the dressing with the type and gauge of the needle used, date, time, and name of the nurse who performed the puncture.	1.00
17. Record the procedure in the patient’s medical chart.	1.00
Maintenance	
18. Always use a 10 ml or larger syringe for catheter manipulation.	1.00
19. Disinfect the connectors before each access or manipulation with an alcohol-based antiseptic solution, applying friction for 5 to 15 seconds.	1.00
20. Maintain the Huber needle for up to seven days, protected by a sterile covering.	0.89
21. Perform heparinization or salinization after the end of use or every 30 days if the catheter is not in use.	1.00
22. When removing the puncture, use 2 ml of the solution (0.2 ml of heparin 5000 IU/ml + 9.8 ml of 0.9% saline) for HEPARINIZATION or 10 ml of 0.9% saline for SALINIZATION using a turbulence technique and then proceed with the removal ofthe needle from the reservoir.	1.00

*RG - Registro Geral (General Registry); CVI: Content Validity Index.*

**Figure 1:**
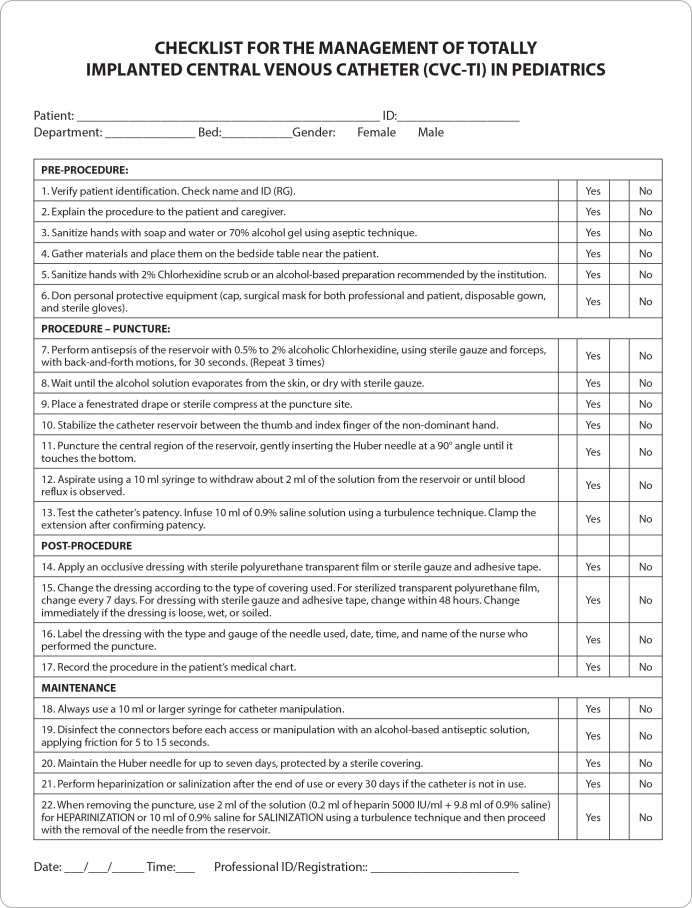
Final version of the Checklist for the Management of Totally Implanted Catheters in Hospitalized Children and Adolescents, 2023

The overall evaluation of the second version of the instrument, described in ten items, presented a global score of 9.9 (Table 2) and the final version of the checklist is presented in Figure 1.

**Table 2:** Overall evaluation of the care checklist for the management of totally implanted catheters in hospitalized children and adolescents by the seven specialists, Uberaba, Minas Gerais, Brazil, 2023

Evaluated Items	Average
Usefulness/Relevance	9.9
Consistency	10
Clarity	9.9
Objectivity	10
Simplicity	9.9
Feasibility	10
Up-to-date Information	9.9
Accuracy	9.9
Instructional Sequence of Topics	9.9
Presentation Format of the Protocol	9.9
Global Evaluation	9.9

## DISCUSSION

Various strategies are used to improve health indicators and promote harm-free care, based on official recommendations and international and national guidelines. One such strategy is the creation of checklists, standardization of procedures, and team training^(19)^. This study considered the checklist as the objective of investigation. In nursing, the construction and validation of instruments guide and direct health practices, providing the development of health technologies, directing nursing care, and improving the quality of care for patient safety. This scientific foundation facilitates the standardization of procedures and team training^(19-20)^. However, gaps related to the level of knowledge and attitudes of nursing professionals regarding the management of CVC-TI are observed^(21)^.

Among the main measures for preventing infection related to CVC-TI are the disinfection of infusion lines and hand hygiene. It is recommended that, before performing invasive procedures (insertion of CVC, punctures, cavity drainage, dialysis installation, minor sutures, endoscopies, and others), hand decontamination should be performed^(22)^. Additionally, all health services in the country must comply with RDC/ANVISA No. 42/2010, which establishes that all health services provide alcohol-based hand rubs in visible and easily accessible locations during care^(22-23)^. Another preventive measure is the adoption of maximal barrier precautions, where the professional must use a surgical mask, cap, sterile gauze, and gloves^(8,24)^.

In the skin preparation phase, the antiseptic of choice is alcoholic chlorhexidine, as it maintains microbicidal action and a residual effect on the skin for a longer duration compared to other substances with the same purpose. It is important to apply it using spiral motions, always from the center outwards, covering the central portion of the reservoir, at least three times^(24)^.

Activation of the device is performed by puncturing the silicone reservoir at a 90° angle with a non-cutting needle (Huber type) until it touches the bottom of the reservoir. Hypodermic needles should not be used due to the risk of perforating the device’s septum^(25)^. If the professional notices resistance or pain during fluid and/or medication infusion, or absence of venous return, the correct position of the needle should be evaluated^(26)^.

The dressing after the puncture should be applied in a sterile manner, and some care is recommended. The replacement time depends on the type of covering used: sterile gauze every 48 hours and sterile semipermeable transparent film every 7 days^(25)^. Regardless of the covering, it should be replaced immediately if it becomes soiled, loose, or wet^(23)^.

The permeability and optimal performance of the device are evaluated by fluid infusion (flow) and blood aspiration (reflux) without resistance. Partial or total occlusion of the CVC-TI is a concerning event, often necessitating the interruption of treatment and sometimes requiring a new invasive procedure to replace the device^(27-28)^.

The patency of the CVC-TI depends on several factors, such as proper washing technique, blocking, and the correct choice of solution, to prevent device occlusion. Flushing the catheters with 0.9% sodium chloride solution after fluid infusion is recommended. Flushing is justified to remove medication residues and to avoid adverse events of incompatibility and occlusions. It is also noted that the use of sterile water for washing catheters is not recommended^(20)^.

Comparative studies on the efficacy of saline versus heparin indicate that 0.9% saline solution is as effective as heparin for catheter locking^(24,27-29)^. The choice between the two depends on clinical criteria, institutional protocols, and the professional’s critical analysis of scientific evidence^(30-31)^. When using heparin or saline solution for catheter removal, positive pressure techniques are recommended for removing and flushing the catheter lumen^(27)^.

After the completion of the therapy, it is recommended to perform catheter locking before removing the needle from the device. For this procedure, heparin solution in various concentrations or saline solution is commonly used to prevent CVC-TI obstruction. Heparin is contraindicated in some situations as it can cause thrombocytopenia, may present incompatibility with the prescribed treatment, and increases the cost of catheter maintenance compared to using 0.9% sodium chloride^(32)^.

Nursing records ensure the quality and continuity of care, legally supporting the actions taken in the profession^(33)^. A study related to the management of totally implanted catheters found a lack of documentation of the care provided by nurses. This same study, through an audit, found that none of the evaluated nurses documented the care provided. After implementing training and adopting a checklist, a subsequent audit showed that 86% of the nurses recorded the care provided in the patient’s chart^(34)^.

Studies on the use of the Surgical Safety Checklist indicate that the tool contributed to improved self-perception of teamwork and communication, bringing additional benefits to the hospital, such as cost reduction through efficiency gains^(35-36)^. The use of the Safe Childbirth Checklist ensures that tasks are performed in the established order, controls the compliance with workplace requirements, and is considered a simple and effective method to reduce potential adverse events^(37-38)^.

It is essential to highlight the need for continuous updates of the team and work processes through educational strategies and training that enable the evaluation of results and foster a culture of institutional safety for healthcare professionals^(8)^. Using instruments to standardize care improves assistance by ensuring the proper execution of specific procedures based on evidence, guiding and encouraging nurses to document and evaluate children with CVC-TI, and, when necessary, adapting these instruments to the characteristics of each service^(23)^.

### Study limitations

The limitations of this study are primarily related to the sample size, particularly the limited number of specialists. However, the number recommended by the literature adopted in the study was met. Another specific challenge was obtaining feedback from the specialists, as their participation is voluntary and requires their availability to engage in the research.

### Contributions to Nursing, Health, or Public Policy

The checklist developed and validated in this study allows hospital health services that care for children and adolescents to evaluate the care provided during the management of CVC-TI, aiming for improvements in care quality. This evidence-based approach utilizes standardized and reliable instruments.

## CONCLUSIONS

This study enabled the construction and content validation of a checklist for the management of CVC-TI in hospitalized children and adolescents, covering four domains: pre-procedure, procedure-puncture, post-procedure, and maintenance, guiding safe steps for quality care in children who require this device. All items were considered valid after the second evaluation round. The validation and implementation of instruments that standardize procedures, in addition to supporting nursing professionals, specifically nurses, during CVC-TI puncture, promote autonomy and quality of care for users of this device. It highlights the importance of conducting further studies on the subject and training the nursing team for proper device management to prevent bloodstream infections and occlusions, thereby avoiding increased hospitalization time for children.
