# Supplementary material for: Construction and validation of a checklist for the management of totally implanted catheters in children
Source: Rev Bras Enferm. 2024 Sep 20;77(4):e20230447. doi: 10.1590/0034-7167-2023-0447 (PMC11419684; doi:10.1590/0034-7167-2023-0447)
Supplement: Translation

# Translation: Construção e validação de lista de checagem para manejo do cateter totalmente implantado em crianças

**DOI:** 10.1590/0034-7167-2023-0447pt

**Authors:** Giselle Vieira de Souza, Isabella Pavarine de Souza, Maria Paula Custodio Silva, Silmara Elaine Malaguti Toffano, Divanice Contim

**Affiliations:** I Universidade Federal do Triângulo Mineiro. Uberaba, Minas Gerais, Brasil; II Universidade Federal do Triângulo Mineiro, Rede Brasileira de Serviços Hospitalares, Hospital de Clínicas. Uberaba, Minas Gerais, Brasil

**Keywords:** Cuidados de Enfermagem, Estudo de Validação, Dispositivos de Acesso Vascular, Enfermagem Pediátrica, Lista de Checagem

## Abstract

**Objetivos:**

construir e validar o conteúdo de uma lista de checagem para manejo do cateter totalmente implantado em crianças e adolescentes hospitalizados.

**Métodos:**

Pesquisa metodológica realizada no período de outubro de 2021 a dezembro de 2022, em duas etapas: elaboração do instrumento com diretrizes para o cuidado e validação de conteúdo da lista de checagem. O instrumento, contendo 23 itens apresentados em formato Likert, foi avaliado online por especialistas em duas rodadas. Aplicou-se o Índice de Validade de Conteúdo, considerando válidos os índices acima de 0,8.

**Resultados:**

a lista final contemplou quatro domínios e 22 itens de checagem, sendo validada com Índice de Validade de 0,98. A avaliação geral do instrumento apresentou uma nota global de 9,9.

**Conclusões:**

a validação e aplicação de instrumentos que padronizam os procedimentos, além de respaldar o profissional, promovem autonomia e qualidade da assistência à criança e ao adolescente em uso deste dispositivo.

## INTRODUÇÃO

De acordo com a Organização Mundial de Saúde (OMS), a cada ano, cerca de 400 mil crianças e adolescentes são diagnosticados com câncer infantil. Trata-se de um grupo etário heterogêneo de doenças raras que surgem entre o nascimento e os 19 anos de idade^(1)^. Os cânceres infantis têm origem em tecidos embrionários que sofrem mutações resultantes da divisão descontrolada de células anormais. Os mais incidentes são a leucemia, associada ao sistema linfático; tumores como neuroblastoma em múltiplos locais, especialmente nas glândulas adrenais, relacionados ao sistema nervoso central; linfoma, ligado a células imunes; tumores renais e tumores ósseos^(2)^.

No Brasil, em 2023, foram registrados aproximadamente 7.930 novos casos de câncer em crianças e adolescentes, observando-se a distribuição do risco estimado de 140,50 casos por milhão para o sexo masculino e 128,87 por milhão para o sexo feminino. Estes são dados relevantes, visto que cerca de 33% da população brasileira é formada por este ciclo vital^(3)^. As neoplasias correspondem à principal causa de óbito em crianças após um ano até o final da adolescência^(3)^.

Os avanços no tratamento do câncer infantil nos últimos 50 anos impactaram de forma significativa, com um aumento de mais de 85% na taxa de sobrevida global^(4-5)^. O desenvolvimento de tecnologias relacionadas ao tratamento do câncer infantil incorporou o cateter venoso central totalmente implantado (CVC-TI) na assistência à saúde, proporcionando melhor qualidade de vida ao paciente. Trata-se de um dispositivo que fornece um acesso venoso para a administração frequente de hemocomponentes, hemoderivados, nutrição parenteral, antibióticos e quimioterápicos em pacientes com necessidade de longas terapias endovenosas (EV)^(6)^.

A inserção do CVC-TI, quando comparado a outros dispositivos, diminui o risco de trombose, reduz o índice de infecção, proporciona maior conforto ao paciente, possibilita tratamento ambulatorial, resguarda o sistema venoso periférico e mantém a rotina de atividades do paciente, diminuindo o estresse e o sofrimento com inúmeras punções sem sucesso^(6)^.

A equipe de enfermagem, sobretudo os enfermeiros, são os principais responsáveis por sua manipulação e manutenção. Conforme normativa do Conselho Federal de Enfermagem (COFEN), que determina a atuação privativa do enfermeiro, compete ao enfermeiro a punção do CVC-TI, administrar quimioterápico, bem como se encarregar dos cuidados com esse dispositivo. Para tanto, o enfermeiro deve ter conhecimento e desenvolver competências quanto ao manejo dos cateteres, atuando com segurança e provendo condutas importantes durante o tratamento de pacientes que necessitam desse dispositivo^(7-8)^.

O manejo adequado do CVC-TI possibilita a diminuição de custos, previne a ocorrência de oclusões e reduz o risco de infecções associadas aos cuidados de saúde. Desse modo, torna-se importante a elaboração de instrumentos para incrementar a segurança e o aperfeiçoamento da qualidade da assistência de enfermagem prestada aos pacientes em uso deste dispositivo^(8)^.

As listas de checagem são ferramentas simples e econômicas que podem ser reproduzidas em diferentes áreas da atenção à saúde. Elas contribuem para a gestão do cuidado, qualificam a assistência prestada e aumentam a segurança dos pacientes internados, oportunizando a prestação de um cuidado seguro e confiável^(9-10)^. Por ser uma ferramenta de trabalho estruturada que contempla um conjunto de itens ou atividades complexas, as listas de checagem são utilizadas para confirmar se as ações e intervenções necessárias na operacionalização de alguma tarefa foram realizadas^(11)^. Estudos apontam que essa ferramenta colabora na melhoria da assistência e possibilita a redução de eventos adversos relacionados à segurança do paciente^(9-11)^.

A importância de elaborar uma lista de checagem para o manejo do cateter totalmente implantado em crianças e adolescentes hospitalizados, a fim de possibilitar uma assistência de qualidade, torna-se evidente. Assim, é necessário responder à seguinte questão: Quais cuidados dispensados ao manejo do cateter venoso central totalmente implantado em crianças e adolescentes hospitalizados devem ser incluídos em uma lista de checagem?

## OBJETIVOS

Construir e validar o conteúdo de uma lista de checagem para manejo do cateter totalmente implantado em crianças e adolescentes hospitalizadas.

## MÉTODOS

### Aspectos éticos

Para atender às exigências da Resolução 466/2012 do Conselho Nacional de Saúde, que regulamenta as normas para a realização de pesquisa envolvendo seres humanos, todos os participantes assinaram o Termo de Consentimento Livre e Esclarecido. O estudo foi aprovado pelo Comitê de Ética em Pesquisa do Hospital de Clínicas da Universidade Federal do Triângulo Mineiro (HC-UFTM), cujo parecer está anexado à presente submissão.

### Tipo e período do estudo

Estudo metodológico desenvolvido no período de março a novembro de 2022, sobre a elaboração e validação de uma lista de checagem, sobre o manejo do CVC-TI em crianças e adolescentes hospitalizadas, em duas fases: elaboração e validação do instrumento analisado por especialistas da área de enfermagem oncológica pediátrica^(12)^. A partir da elaboração do instrumento ocorreu o processo de validação por meio de um painel online de especialistas utilizando-se da estratégia combinada da validação de conteúdo e obtenção de consenso^(13)^.

### População e amostra

Para a etapa de validação do conteúdo foram selecionados enfermeiros da área de enfermagem pediátrica, por meio de uma consulta no Currículo Lattes, com ativação do filtro de atuação profissional. Foram selecionados sessenta e cinco enfermeiros que atenderam ao critérios de Fehring^(14)^.

Foi estabelecido um prazo de 15 dias para a devolução dos instrumentos de avaliação. Nesse momento foram excluídos os que informaram motivos que impedissem a participação no estudo. Quatorze retornaram o instrumento preenchido na primeira rodada de avaliação. Na segunda rodada foram realizadas as adequações sugeridas e o instrumento foi encaminhado via *e-mail* para os quatorze especialistas da primeira rodada e obteve-se retorno de sete avaliações.

### Procedimentos metodológicos

O instrumento foi construído pelas pesquisadoras com base no Procedimento Operacional Padrão (POP) sobre a Punção do Cateter Totalmente Implantado do HC-UFTM^(15)^ o caderno Medidas de Prevenção de Infecção Relacionada à Assistência à Saúde da Agência Nacional de Vigilância Sanitária(ANVISA)^(16)^; e um estudo sobre Heparinização e Salinização do cateter^(17)^.

A primeira versão da lista de checagem foi estruturada com base em quatro domínios: Pré - procedimento, Procedimento - punção, Pós - procedimento e Manutenção. A lista de checagem nomeada Lista para manejo do cateter totalmente implantado em crianças e adolescentes hospitalizados, foi elaborada em padrão *HyperText Markup Language* (HTML) no *Google Forms* ^®^.

Os especialistas receberam uma carta convite via e-mail, com explicações dos objetivos e procedimentos do estudo e solicitação de resposta para participar do estudo. Após essa fase os potenciais participantes receberam por meio da plataforma *Google Forms*: Termo de Consentimento Livre e Esclarecido, e após sua assinatura receberam o link para acessar um formulário de caracterização sociodemográfica incluindo sexo, idade, tempo de atuação na área e formação acadêmica, tempo de atuação na área de enfermagem oncológica pediátrica e lista de checagem a ser validada.

Para avaliar cada itens elencado utilizou-se a escala de *Likert* de cinco pontos ordinais, sendo que: (cinco) equivale a concordo totalmente, (quatro) equivale à concordo, (três) equivale à nem concordo/nem discordo, (dois) equivale a discordo, (um) equivale a discordo totalmente, foi acrescido um espaço para respostas descritivas opcionais. Para análise geral do instrumento considerou-se os requisitos: utilidade/pertinência, consistência, clareza, objetividade, simplicidade, exequibilidade, atualização, vocabulário, precisão, sequência instrucional dos tópicos; ao fim, a nota global^(13)^.

### Análise do estudo e estatística

Os dados obtidos foram importados do *Google Forms* ^®^ para um banco de dados do *Microsoft Excel* ^®^, e analisados com base no Índice de Validade de Conteúdo (IVC), para medir a proporção ou porcentagem de especialistas que concordam com determinados aspectos do instrumento e de seus itens, permitindo analisar cada item de forma individualizada e o instrumento como um todo. O IVC considerado válido para este estudo foi acima de 0,8^(18)^, às adequações e sugestões da primeira rodada, foram incluídas e submetidas na segunda rodada de avaliação. O IVC por item (IVC-I) foi calculado por meio do somatório de concordância dos itens que foram classificados como três ou quatro. Os itens que receberam pontuação um ou dois foram revisados ou eliminados.

## RESULTADOS

Do total de especialistas que aceitaram participar da pesquisa, 14 fizeram parte da amostra da primeira rodada e sete da segunda rodada de validação. Todas eram enfermeiras do sexo feminino, com média de idade de 40 anos, variando entre 28 e 64 anos. Entre elas, 14% eram mestres e 86% doutoras. Além disso, 64% eram docentes na área de enfermagem pediátrica e 26% trabalhavam em hospitais federais, com tempo médio na área de oncologia pediátrica ou materno-infantil de 6 anos. No total, 78% possuíam curso de manejo do cateter e 85% realizavam punções do CVC-TI.

A primeira versão encaminhada tinha 23 itens, subdivididos em quatro domínios: Pré-procedimento, Procedimento - punção, Pós-procedimento e Manutenção. Todos os itens foram avaliados segundo a escala Likert. As sugestões foram consideradas e as alterações necessárias foram realizadas.

Em todos os itens, a concordância estava dentro do nível estabelecido (IVC > 0,8). As sugestões foram consideradas, as alterações foram realizadas, e o instrumento foi encaminhado para uma nova rodada. Entre as sugestões, duas questões (6 e 7) foram fundidas, resultando em um instrumento final com 22 itens. A segunda rodada foi utilizada para realizar ajustes no instrumento validado, e seu IVC está descrito na Tabela 1.

**Tabela 1:** Segunda rodada de validação de conteúdo da lista de cuidados para o manejo de cateter totalmente implantado em crianças e adolescentes hospitalizadas pelos sete especialistas, considerando o Índice de Validade de Conteúdo, Uberaba, Minas Gerais, Brasil, 2023

Manejo de cateter totalmente implantado em crianças hospitalizadas	IVC
Pré - procedimento	
1 - Conferir identificação do paciente. Checar nome e RG.	1,00
2 - Explicar o procedimento para o paciente e o responsável.	1,00
3 - Higienizar as mãos com água e sabão ou fricção com álcool gel 70% em técnica asséptica.	1,00
4 - Reunir os materiais e posicioná-los em mesa de utilidades móvel, próximo ao leito do paciente.	1,00
5 - Higienizar as mãos com clorexidina degermante 2% ou fricção com preparação à base de álcool preconizada pela instituição.	0,97
6 - Realizar paramentação (gorro, máscara cirúrgica - profissional e paciente, avental descartável e luva estéril)	0,91
Procedimento - punção	
7 - Realizar antissepsia do reservatório com clorexidina alcoólica 0,5 a 2%, com auxílio de gaze e pinça estéril, técnica em Z, por 30 segundos. (Repetir por 3x)	0,89
8 - Aguardar até que a solução alcóolica evapore da pele, ou realizar a secagem com gaze estéril.	0,89
9 - Posicionar campo fenestrado ou compressa estéril no local de punção.	1,00
10 - Estabilizar o reservatório do cateter entre os dedos indicador e polegar da mão não dominante.	1,00
11 - Puncionar a região central do reservatório, inserindo DELICADAMENTE a agulha (tipo *hubber*) em ângulo de 90°, até tocar a sua parte inferior.	1,00
12 - Aspirar utilizando seringa de 10 ml, a solução contida no reservatório, cerca de 2ml ou até observar refluxo de sangue.	1,00
13 - Testar a perviedade do cateter. Infundir 10ml de SF0,9% em técnica de turbilhonamento. Clampear a extensão após confimar a perviedade.	1,00
Pós - procedimento	
14 - Realizar curativo oclusivo com filme transparente de poliuretano estéril ou gaze estéril e fita adesiva.	1,00
15 - Trocar o curativo de acordo com a cobertura utilizada. Para Filme transparente de poliuretano esterilizado trocar a cada 7 dias. Para curativo com gaze estéril e fita adesiva trocar em até 48h. Troca imediata se curativo solto, úmido ou com sujidade.	1,00
16 - Realizar a identificação do curativo com o tipo e calibre da agulha utilizada, data, horário e nome do enfermeiro que realizou a punção.	1,00
17 - Registrar o procedimento no prontuário do paciente.	1,00
Manutenção	
18 - Utilizar sempre seringa de 10ml ou acima, para manipulação do cateter.	1,00
19 - Realizar desinfecção dos conectores antes de cada acesso ou manipulação com solução antisséptica a base de álcool, com movimentos aplicados de forma a gerar fricção mecânica, de 5 a 15 segundos.	1,00
20 - Manter a agulha *Hubber* por até sete dias, protegida por cobertura estéril.	0,89
21 - Realizar heparinização ou salinização após o término do uso ou a cada 30 dias sem utilização do cateter.	1,00
22 - Com indicação de retirada da punção, utilizar 2ml da solução (0,2ml de heparina 5000UI/ml + 9,8ml de SF0,9%) para HEPARINIZAÇÃO OU 10ml de SF0,9% para SALINIZAÇÃO em técnica de turbilhonamento e proceder com a retirada da agulha do reservatório.	1,00

*RG - Registro Geral; IVC - Índice de Validade de Conteúdo.*

A avaliação geral da segunda versão do instrumento, descrita em dez itens, apresentou uma nota global de 9,9 (Tabela 2) e a versão final da lista de checagem, apresentada na Figura 1.

**Tabela 2:** Avaliação geral da lista de cuidados para o manejo de cateter totalmente implantado em crianças e adolescentes hospitalizadas pelos sete especialistas, Uberaba, Minas Gerais, Brasil, 2023

Itens avaliados	Média
Utilidade/pertinência	9,9
Consistência	10
Clareza	9,9
Objetividade	10
Simplicidade	9,9
Exequibilidade	10
Atualização	9,9
Precisão	9,9
Sequência instrucional dos tópicos	9,9
Forma de apresentação do protocolo	9,9
Avaliação Global	9,9

**Figura 1:** Versão final da lista para manejo do cateter totalmente implantado em crianças e adolescentes hospitalizados, 2023

## DISCUSSÃO

Diversas estratégias são utilizadas para melhorar indicadores em saúde e promover um cuidado isento de danos na assistência, embasadas em recomendações oficiais e diretrizes internacionais e nacionais. Uma dessas estratégias é a criação de listas de checagem, padronização de procedimentos e treinamentos das equipes^(19)^. A lista de checagem foi considerada neste estudo como objetivo de investigação. Na enfermagem, a construção e validação de instrumentos orientam e norteiam as práticas de saúde, proporcionando o desenvolvimento de tecnologias de saúde, direcionando os cuidados de enfermagem e melhorias na qualidade da assistência para a segurança do paciente, embasando cientificamente o profissional e facilitando a padronização de procedimentos e treinamentos das equipes^(19-20)^. No entanto, são observadas lacunas relacionadas ao nível de conhecimentos e às atitudes dos profissionais de enfermagem quanto ao manejo do CVC-TI^(21)^.

Dentre as principais medidas para a prevenção de infecção relacionada ao CVC-TI estão a desinfecção das linhas de infusão e a higienização das mãos. É preconizado que, antes da realização de procedimentos invasivos (inserção de CVC, punções, drenagens de cavidades, instalação de diálise, pequenas suturas, endoscopias e outros), seja realizada a degermação das mãos^(22)^. Além disso, todos os serviços de saúde do país devem se adequar à RDC/ANVISA nº 42/2010, a qual estabelece que todos os serviços de saúde disponibilizem preparações alcoólicas para fricção antisséptica das mãos em locais visíveis e de fácil acesso durante a assistência^(22-23)^. Outra forma de prevenção é adotar barreira máxima, onde o profissional deve utilizar máscara cirúrgica, gorro, gaze e luva estéril^(8,24)^.

Na fase de preparo da pele, o antisséptico de escolha é a clorexidina alcoólica, pois mantém ação microbicida, além de manter efeito residual na pele, com tempo superior comparado a outras substâncias com a mesma finalidade, destacando a importância de ser realizada através de movimentos em espiral, sempre de dentro para fora, partindo da porção central do reservatório, por pelo menos três vezes^(24)^.

A ativação do dispositivo é realizada por meio de punção do reservatório de silicone, em ângulo de 90º com agulha não cortante (tipo *Huber*) até tocar o fundo do reservatório. Não devem ser usadas agulhas hipodérmicas pelo risco de perfuração do septo do dispositivo^(25)^. Caso o profissional note resistência ou dor à infusão de fluidos e/ou medicações, ou ausência de retorno venoso, deve-se avaliar a posição correta da agulha^(26)^.

O curativo após a punção deve ser realizado de modo estéril e alguns cuidados são recomendados. O tempo de troca é dependente do tipo de cobertura escolhida: com gaze estéril a cada 48 horas e a cada 7 dias com filme transparente semipermeável estéril^(25)^. Independentemente da cobertura, a troca deve ser imediata se observada sujidade, estiver solto ou apresentar umidade^(23)^.

A permeabilidade e o desempenho ideal do dispositivo são avaliados por meio da infusão de fluidos (fluxo) e da aspiração de sangue (refluxo) sem oferecer resistência. A oclusão parcial ou total do CVC-TI é um acontecimento preocupante, visto que, frequentemente, é necessária a interrupção do tratamento e, em algumas vezes, a realização de um novo procedimento invasivo para a substituição do dispositivo^(27-28)^.

A patência do CVC-TI depende de alguns cuidados, como técnica adequada de lavagem, bloqueio e escolha da solução correta, prevenindo a oclusão do dispositivo. Recomenda-se a realização do flushing nos cateteres com solução de cloreto de sódio a 0,9% após a infusão de fluidos. O flushing justifica-se para a retirada de resíduos de medicações, com o intuito de evitar eventos adversos de incompatibilidade e oclusões. Ressalta-se ainda que não é recomendado o uso de água estéril para lavar os cateteres^(20)^.

Estudos comparativos sobre a eficácia da solução salina versus heparina sustentam que a solução salina 0,9% é tão eficaz quanto a heparina como solução de bloqueio do cateter^(24,27-29)^. A escolha entre uma ou outra depende dos critérios clínicos, protocolos institucionais e análise crítica do profissional frente às evidências científicas^(30-31)^. Ao adotar o uso de heparina ou solução salina na remoção do cateter, recomenda-se a utilização de técnicas de pressão positiva para a remoção e a lavagem do lúmen do cateter^(27)^.

Após a finalização da terapêutica, é preconizado o bloqueio para posterior retirada da agulha do dispositivo. Para esse procedimento, é comumente utilizada a solução de heparina em diversas concentrações ou solução fisiológica, como forma de prevenção de obstrução do CVC-TI. A heparina é contraindicada em algumas situações por ocasionar trombocitopenia, podendo apresentar incompatibilidade com o tratamento prescrito, além de elevar o custo de manutenção do cateter em comparação ao uso de cloreto de sódio 0,9%^(32)^.

Os registros de enfermagem asseguram a qualidade e a continuidade da assistência, respaldando legalmente as ações realizadas no exercício da profissão^(33)^. Estudo relacionado ao manejo do cateter totalmente implantado verificou ausência de registros da assistência prestada pelo enfermeiro. Esse mesmo estudo, por meio de auditoria, constatou que nenhum dos enfermeiros avaliados realizava a documentação da assistência. Após a implementação de treinamento e adoção de lista de checagem, houve nova verificação e 86% dos enfermeiros registraram os cuidados realizados em prontuário^(34)^.

Estudos sobre o uso da Lista de Verificação de Segurança Cirúrgica abordam que o uso da ferramenta contribuiu para a melhoria da autopercepção do trabalho em equipe e comunicação, trazendo ainda benefícios para o hospital, como redução de custos por meio de ganhos de eficiência^(35-36)^. O uso da lista de verificação para Partos Seguros proporciona que as tarefas ocorram na ordem estabelecida, controlando o cumprimento de requisitos do ambiente de trabalho, sendo considerado um método simples e eficaz para reduzir possíveis eventos adversos^(37-38)^.

Ressalta-se a necessidade de atualização contínua da equipe e dos processos de trabalho, por meio de estratégias educativas e treinamento que possibilitem avaliação dos resultados e cultura de segurança institucional para o profissional de saúde^(8)^. A utilização instrumentos com intuito de padronizar o cuidado, melhora a assistência ao possibilitar a realização adequada dos procedimentos específicos, baseados em evidências, direcionando e incentivando os enfermeiros a documentarem e avaliar as crianças portadoras de CVC-TI e, quando necessário adaptar estes instrumentos às características de cada serviço^(23)^.

### Limitações do estudo

As limitações deste estudo concentram-se na amostra, sobretudo no número limitado de especialistas. No entanto, foi contemplado o número recomendado pela literatura adotada no estudo. Outra dificuldade específica foi obter o retorno dos especialistas, uma vez que sua contribuição é voluntária e exige disponibilidade por parte deles para a participação na pesquisa.

### Contribuições para a área da enfermagem, saúde ou política pública

A lista de checagem elaborada e validada neste estudo possibilita aos serviços de saúde hospitalares que atendem crianças e adolescentes avaliarem a assistência prestada durante o manejo do CVC-TI, visando melhorias na qualidade da assistência, em um cuidado baseado em evidências por meio de instrumentos padronizados e confiáveis.

## CONCLUSÕES

Este estudo possibilitou a construção e a validação de conteúdo da lista de checagem para o manejo do CVC-TI em crianças e adolescentes hospitalizados, com quatro domínios: pré-procedimento, procedimento-punção, pós-procedimento e manutenção, norteando etapas seguras para um cuidado de qualidade em crianças que necessitam deste dispositivo. Todos os itens foram considerados válidos após a segunda rodada de avaliação. A validação e implementação de instrumentos que padronizem os procedimentos, além de respaldar o profissional de enfermagem, em específico o enfermeiro, durante a punção do CVC-TI, promovem autonomia e qualidade da assistência ao usuário deste dispositivo. Ressalta-se a importância da realização de novos estudos sobre o assunto e a capacitação da equipe de enfermagem para o manejo adequado do dispositivo, de maneira a prevenir a incidência de infecções de corrente sanguínea e oclusões, evitando assim o aumento do tempo de hospitalização da criança.
